# Transient hepatic intensity differences: correlations with treatment outcomes and adverse events following DEB-TACE in hepatocellular carcinoma

**DOI:** 10.1186/s13244-025-02041-2

**Published:** 2025-08-01

**Authors:** Noble Chibuike Opara, Shuwei Zhou, Weilang Wang, Shuhang Zhang, Xunjun Chen, Shenghong Ju, Yuan-Cheng Wang

**Affiliations:** 1 Department of Radiology, Zhongda Hospital, Nurturing Center of Jiangsu Province for State Laboratory of AI Imaging & Interventional Radiology, School of Medicine, Southeast University, Nanjing, China; 2 Department of Radiology, The Peoples Hospital of Xuyi County, Huaian, China

**Keywords:** Transient hepatic intensity differences, Drug-eluting bead transarterial chemoembolization, Hepatocellular carcinoma, Overall survival, Progression-free survival

## Abstract

**Background:**

Transient hepatic intensity differences (THID) on MRI are commonly observed in hepatocellular carcinoma (HCC) patients following drug-eluting bead transarterial chemoembolization (DEB-TACE). We evaluated the association between THID, treatment outcomes, and adverse events in HCC patients treated with DEB-TACE.

**Materials and methods:**

This retrospective analysis included data from a prospective study conducted with 102 consecutive HCC patients treated with DEB-TACE between December 2017 and December 2020. The chi-square test assessed correlations between THID and adverse events, including biliary injury, intrahepatic metastasis, and portal venous thrombosis. Kaplan–Meier method evaluated overall survival (OS) and progression-free survival (PFS), with log-rank tests comparing THID complexity (simple vs complex) and severity (mild, moderate, and severe). Logistic regression identified factors associated with THID development.

**Results:**

Among the 102 HCC patients, 74 (72.5%) developed THID after DEB-TACE. Patients with THID had significantly higher rates of biliary injury (47% vs 14.3%, *p* = 0.002) and intrahepatic metastasis (25.7% vs 7.1%, *p* = 0.030). Complex THID was associated with worse PFS (*p* = 0.024). Moderate-to-severe THID had worse OS (*p* = 0.019) and PFS (*p* = 0.038). Factors associated with THID development included a higher tumor burden, baseline THID, and Child–Pugh class A.

**Conclusion:**

THID correlates with an increased risk of biliary injury and intrahepatic metastasis and is associated with worse OS and PFS in HCC patients following DEB-TACE.

**Critical relevance statement:**

THID development after DEB-TACE correlates with higher incidence of biliary injury, intrahepatic metastasis, and worse OS/PFS, emphasizing its potential as a critical imaging biomarker. This study advances clinical radiology by highlighting THID as an important factor in treatment outcomes.

**Key Points:**

- THID after drug-eluting bead transcatheter arterial chemoembolization correlates with treatment outcomes and adverse events in HCC.
- THID correlates with higher biliary injury, intrahepatic metastasis, and reduced survival rates.
- Complex or severe THID correlates with poorer survival in HCC patients.

**Graphical Abstract:**

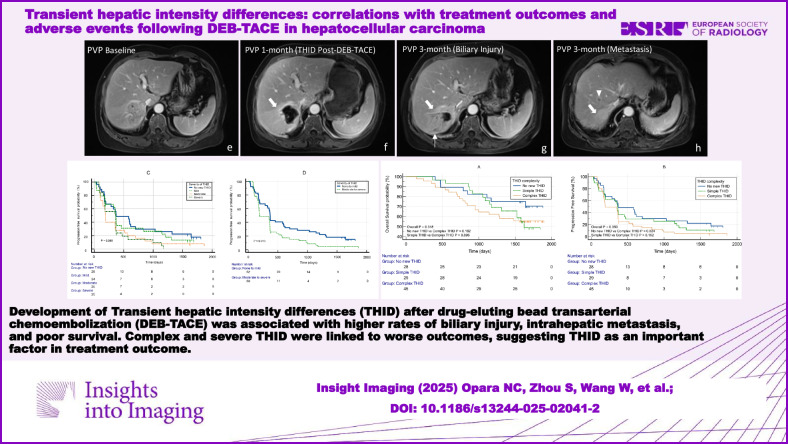

## Introduction

Hepatocellular carcinoma (HCC) is a leading cause of cancer-related mortality worldwide, with many patients diagnosed at advanced stages, often limiting their eligibility for curative treatments, including surgery or liver transplantation [1]. Consequently, local regional therapies, including conventional transarterial chemoembolization (cTACE) and Drug-eluting bead transcatheter arterial chemoembolization (DEB-TACE), have become essential in managing unresectable HCC and improving survival outcomes [2–4].

Transient hepatic intensity differences (THID), are perfusion-related changes in liver parenchyma [5, 6] and are frequently observed on imaging after TACE [7, 8] and are common in HCC patients. These differences in signal intensity reflect dynamic alterations in blood flow and vascularity within the liver, particularly during the arterial phase. The liver imaging reporting and data system treatment response algorithm (LI-RADS TRA) assesses treatment response by evaluating not only the tumor but also perfusion changes within the liver parenchyma around the treated lesion, particularly noting perilesional enhancement [9–11]. This study extends the analysis by evaluating THID not only in the perilesional area but also throughout the liver parenchyma. While THID is commonly observed, its clinical significance, particularly its correlation with OS, PFS, and adverse events, remains unclear.

This study aims to explore the relationship between the complexity and severity of THID, treatment outcomes, and adverse events in HCC patients treated with DEB-TACE.

## Materials and methods

This retrospective analysis was performed using data from a prospective, single-arm, multicenter trial assessing the safety and efficacy of DEB-TACE for HCC. Conducted across ten clinical sites in China from October 2017 to December 2020, the study was approved by each center’s Institutional Review Board/Ethics Committee (Identifier: 2017ZDSYLL022-P01) and registered on ClinicalTrials.gov (Identifier: NCT03113955). All participants provided written informed consent, ensuring voluntary participation and compliance with ethical guidelines per the Declaration of Helsinki and local regulations [12].

### Study population

**Fig. 1:**
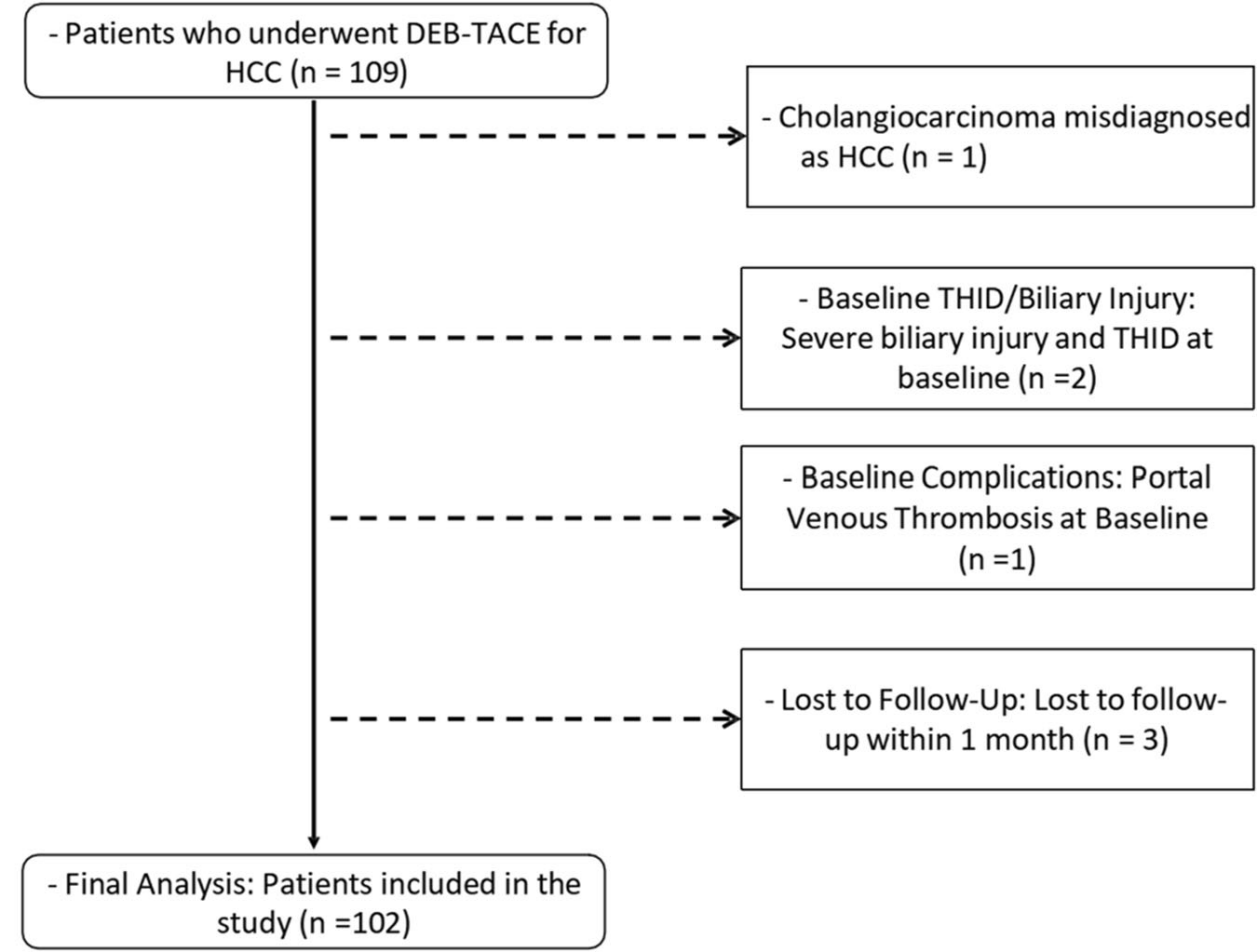
Flowchart of patient selection. THID, transient hepatic intensity differences; DEB-TACE, drug-eluting beads transarterial chemoembolization; HCC, hepatocellular carcinoma

Patients diagnosed with HCC and treated with DEB-TACE were included in the study (Fig. 1). A comprehensive baseline evaluation was conducted, including physical examination, demographic data, tumor and treatment history, Eastern cooperative oncology group performance status (ECOG PS), Child–Pugh score, multi-phase MRI (Supplementary Tables S1 and S2) for evaluating intrahepatic lesions, and laboratory tests for renal and hepatic function, coagulation, hepatitis serology, and alpha-fetoprotein levels. Inclusion criteria included patients aged ≥ 18 who underwent DEB-TACE as their first treatment (treatment-naïve), with at least 3 months of post-treatment MRI follow-up (90 ± 14 days), and Child–Pugh class A or B. Exclusion criteria included baseline portal venous thrombosis, severe biliary injury (defined as the presence of more than one biliary stricture with greater than 50% luminal narrowing, and biloma larger than 2 cm in diameter), severe THID (defined as parenchymal enhancement involving multiple liver segments, with a cumulative total exceeding 3 cm in diameter), or loss to follow-up within 1 month.

### Imaging analysis

Two radiologists (N.C.O. and S.Z.; 4 years and 5 years of experience in abdominal imaging, respectively) independently reviewed multiphase MRI images. Discrepancies in categorizing viable tumors or imaging features were resolved by a third reviewer (Y.C.W., with 13 years of liver imaging experience) to establish a consensus. The definitions of each feature are summarized in Supplementary Table S3. The primary focus of the MRI analysis was the evaluation of THID, characterized by abnormal enhancement of the liver parenchyma relative to surrounding tissues, particularly evident during the arterial phase. THID manifests as regions of altered signal intensity within the liver compared to adjacent tissue. These transient changes may be associated with focal lesions or underlying liver disorders, potentially reflecting persistent vascular or hepatic abnormalities. However, it is important to note that THID can also represent a normal physiological response to treatment or transient hemodynamic changes [13–16].

**Fig. 2:**
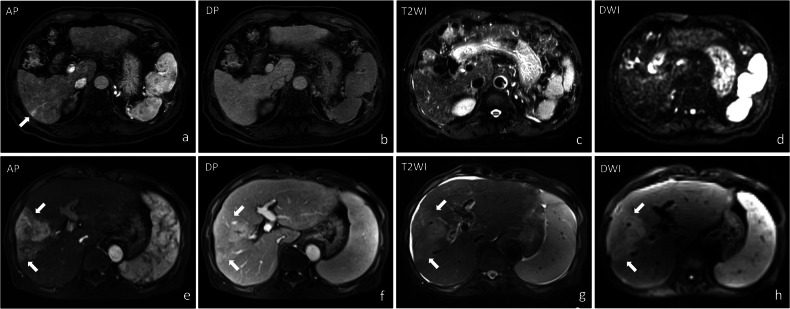
Complexity of THID Following DEB-TACE. Simple THID (**a**–**d**): **a** axial T1-weighted image in the arterial phase shows mild, transient abnormal enhancement in Segment 6 (arrow). The enhancement resolves in subsequent imaging sequences, as shown in the delayed phase (**b**), T2-weighted imaging (**c**), and diffusion-weighted imaging (**d**). Complex THID (**e**–**h**): **e** axial T1-weighted image in the arterial phase illustrates persistent abnormal parenchymal enhancement in Segment 8 (arrow). **f** Axial T1-weighted image in the delayed phase, **g** T2-weighted imaging (T2WI), and **h** diffusion-weighted imaging (DWI) all demonstrate hyperintensity in the affected region

**Fig. 3:**
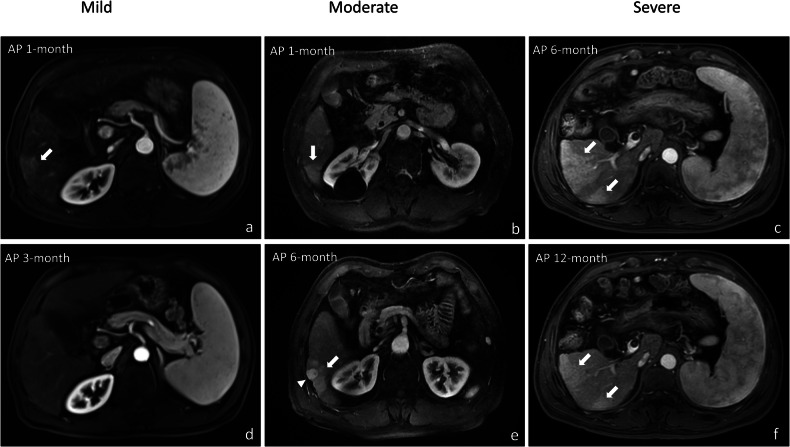
Severity of THID Following DEB-TACE. Mild THID (**a**, **d**): **a** axial T1-weighted image in the arterial phase shows abnormal parenchymal enhancement in Segment 6 (arrow) at 1 month post-DEB-TACE, which resolves by the 3-month follow-up (**d**). Moderate THID (**b**, **e**): **b** axial T1-weighted image in the arterial phase shows abnormal enhancement in Segment 6 (arrow) observed at 1 and 6 months post-DEB-TACE. **e** By 6 months, increased intensity of enhancement is noted surrounding a new metastatic lesion (arrowhead) in S6. Severe THID (**c**, **f**): **c** axial T1-weighted image in the arterial phase shows abnormal parenchymal enhancement involving Segments 5 and 6 (arrows) at 6 months post-DEB-TACE, with persistent but diminished enhancement evident at the 12-month follow-up (**f**)

In this study, a ‘THID event’ was defined as the emergence of new THID or the progression of existing ones, as evidenced by dynamic signal changes in liver parenchyma observed between MRI time points. These changes include the appearance of new areas of parenchymal enhancement or an increase in the intensity or size of previously identified areas. Post-DEB-TACE THID complexity was categorized into simple and complex THID (Fig. 2). Simple THID was defined as abnormal parenchymal enhancement in the arterial phase, with normalization in subsequent phases. Complex THID involved abnormal parenchymal enhancement in the arterial phase along with at least one additional abnormality, including hyper- or hypo-intensity in the portal venous or delayed phases (PVP/DP), or hyperintense signals on T2-weighted imaging (T2WI) and diffusion-weighted imaging (DWI). Patients with both simple and complex THID at different time points were classified as having complex THID. THID severity was graded based on the number of THID events observed: mild (1 event), moderate (2 events), or severe (≥ 3 events, or any single event exceeding 3 cm in maximum diameter, or involving multiple segments with a cumulative total exceeding 3 cm) (Fig. 3). These definitions for simple and complex THID, as well as THID severity, were established by our research team and are specific to this study.

### Treatment protocol and follow-up assessment

The DEB-TACE procedure was standardized and performed by experienced interventional radiologists at each center, using cone-beam CT guidance. Tandem Microspheres loaded with Epirubicin size (40 μm, 75 μm, or 100 μm) and dosage (mL) were tailored to each patient based on safety considerations and tumor characteristics, including size and the risk of vascular shunting. Tumors larger than 5 cm or those with a high risk of shunting were treated with 100 μm microspheres, while tumors smaller than 3 cm with low shunting risk received 40 μm microspheres. Tumors of intermediate size typically received 75 μm microspheres [17].

Effectiveness and side effects were assessed during the procedure and at follow-up visits scheduled for 30 days, 3 months, 6 months, and 1 year. A post-treatment evaluation was conducted every 30 days to determine whether additional procedures were necessary. If required, subsequent treatments were administered within 14 days. Patients could undergo up to four DEB-TACE sessions within a 6-month period. After the fourth treatment, the 30-day follow-up was omitted, and the next visit took place 6 months after the initial treatment.

Follow-up assessments included physical examination, Child–Pugh score, ECOG PS, laboratory tests for hepatic, renal, and coagulation function, and contrast-enhanced MRI to evaluate intrahepatic tumors. Treatment response was measured using modified response evaluation criteria in solid tumors (mRECIST), and any adverse events since the last evaluation were documented.

### Study outcome

Treatment response was assessed using the mRECIST on follow-up MRIs. The objective response rate (ORR) represented the percentage of patients with complete or partial responses. The primary outcome, overall survival (OS), was defined as the time from the first TACE to death from any cause. The secondary outcome, progression-free survival (PFS), measured the time from the latest treatment to disease progression or death.

### Statistical analysis

Continuous variables were analyzed using the Mann–Whitney *U*-test and presented as median (25th–75th percentile). The chi-square test (χ²) compared categorical variables between the THID and non-THID groups, with results presented as frequency and percentage. Logistic regression identified factors associated with THID following DEB-TACE. Variables with *p* < 0.05 in univariate analysis were included in multivariate logistic regression to determine independent predictors, with odds ratios (ORs) and 95% confidence intervals (CIs) estimated. Survival analysis for OS and PFS was performed using the Kaplan–Meier method, with log-rank tests comparing groups based on THID complexity (simple vs complex) and severity (none-to-mild vs moderate-to-severe). None-to-mild THID included patients with no or mild THID, while moderate-to-severe THID included patients with moderate or severe THID. Interobserver agreement was assessed using unweighted and weighted Kappa: κ < 0.00 (poor), 0.00–0.20 (slight), 0.21–0.40 (fair), 0.41–0.60 (moderate), 0.61–0.80 (substantial), and 0.81–1.00 (almost perfect). Partial correlation tests assessed the relationship between THID and adverse events, adjusting for potential confounders. All analyses were performed using IBM SPSS Statistics version 23.0 (IBM Corp.).

## Results

A flowchart of the study is shown in Fig. 1. Initially, 109 patients with HCC were considered for enrollment. Seven patients were excluded based on the following criteria: 1 patient was misdiagnosed with HCC instead of cholangiocarcinoma, 3 patients were lost to follow-up within the first month, 1 patient had portal venous thrombosis at baseline, and 2 had severe biliary injury and THID at baseline. Finally, 102 patients with HCC were included in the final analysis. Among these, 74 patients (72.5%) developed new THID (60 men; median age 62 years, IQR 55–67 years). The baseline characteristics of these patients are summarized in Table 1.

**Table 1:** Baseline clinical and THID characteristics of patients

Variables	New THID (*n* = 74)	No new THID (*n* = 28)	*p* value
Age (years)	62 (55–67)	61 (53–67)	0.750
Sex (male)	60 (81.1)	19 (67.9)	0.124
Diabetes	11 (14.9)	4 (14.3)	0.607
Hypertension	20 (27.0)	6 (21.4)	0.380
Cirrhosis	52 (70.3)	21 (75)	0.417
Baseline THID	29 (39.2)	4 (14.3)	0.013*
Biliary dilatation	11 (14.9)	2 (7.14)	0.246
Number of lesions	1 (1–2)	1 (1–1)	0.006*
BCLC stage 0/A/B	11/39/24	4/23/1	0.007*
Child–Pugh class A	69 (93.2)	22 (78.6)	0.043*
Tumor diameter (cm)^†^	3 (2–5)	3 (2–5)	0.961
DEB-TACE characteristics			
Micro-sphere diameter (μm)	150 (75–190)	115 (75–150)	0.259
DEB-TACE sessions	2 (1–2)	2 (1–2)	0.431
Cumulative dose (mL)	17 (9–25)	12 (4–20)	0.034*
Laboratory test			
Total bilirubin (µmol/L)	15 (10–20)	21 (13–26)	0.015*
Aspartate aminotransferase (U/L)	28 (21–39)	32 (23–43)	0.172
Alanine aminotransferase (U/L)	29 (19–39)	28 (18–41)	0.807
Alkaline phosphatase (U/L)	86 (70–86)	90 (71–114)	0.601
Albumin (g/L)	37 (4–42)	39 (31–44)	0.485
Total protein (g/L)	69 (65–74)	70 (63–76)	0.863
α-Fetoprotein level (µg/L)	69 (4–188)	11 (4–36)	0.433
Prothrombin time (s)	12 (12–14)	13 (12–15)	0.028*

Unless stated otherwise, data are presented as numbers (percentages) or median (25–75 percentiles) as appropriate

*THID* transient hepatic intensity differences, *DEB-TACE* drug-eluting bead transarterial chemoembolization, *BCLC* Barcelona clinic liver cancer

* *p* values indicate a significant difference (*p* < 0.05)

^†^ For patients with multiple lesions, the sum of the longest diameters of all measurable lesions was calculated to represent the total tumor diameter according to mRECIST criteria

There were no significant differences in age, sex, or the prevalence of conditions like diabetes, hypertension, cirrhosis, and tumor size between the two groups. A significantly higher proportion of patients with new THID had a history of baseline THID (39.2% vs 14.3%, *p* = 0.013). The new THID group also had a more advanced Barcelona clinic liver cancer (BCLC) stage distribution (BCLC, 11/39/24 vs 4/23/1, *p* = 0.007) and was more likely to have Child–Pugh class A (93.2% vs 78.6%, *p* = 0.043). Additionally, the new THID group had a higher median number of lesions (1 [1, 2] vs 1 [1], *p* = 0.006) and higher cumulative treatment drug doses (17 [9–25] vs 12 [4–20], *p* = 0.034). However, this group showed lower total bilirubin levels (15 [10–20] vs 21 [13–26], *p* = 0.015) and shorter prothrombin time (12 [12–14] vs 13 [12–15], *p* = 0.028).

**Fig. 4:**
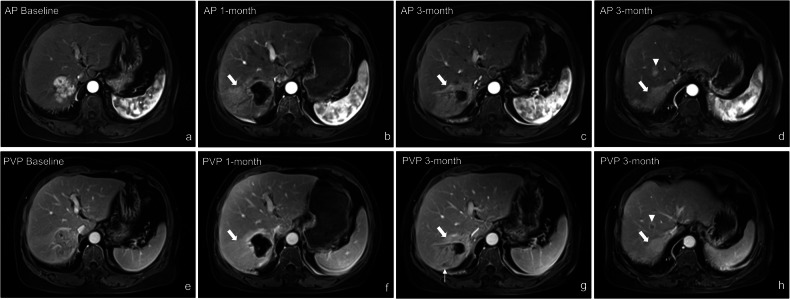
Imaging progression showing the association between THID, biliary injury, and intrahepatic metastasis in a 58-year-old male patient with HCC treated with drug-eluting bead transarterial chemoembolization (DEB-TACE). **a**, **e** Baseline axial T1-weighted images in the arterial phase (AP) (**a**) and portal venous phase (PVP) (**e**) show a HCC lesion in Segment 7. **b**, **f** One-month follow-up MRI reveals the development of THID in Segment 7 associated with an arterioportal shunt (APS) (arrow) visible in both the arterial phase (**b**) and portal venous phase (**f**). **c**, **d**, **g**, **h** Three-month follow-up MRI demonstrates subsequent complications, with biliary injury (straight arrow) visible in the portal venous phase (**g**) and an intrahepatic metastatic lesion (arrowhead) identified in Segment 7 on both the arterial phase (**d**) and portal venous phase (**h**)

Regarding treatment outcomes, when comparing patients with new THID (*n* = 74) to those without (*n* = 28), no significant differences were found in complete response (CR) (*p* = 0.189) or partial response (PR) (*p* = 0.775). However, stable disease (SD) was significantly lower in the new THID group (6.8% vs 21.4%, *p* = 0.033), while progressive disease (PD) was more common (41.9% vs 10.7%, *p* = 0.002). The disease control rate (DCR: CR + PR + SD) was significantly lower in the new THID group (56.8% vs 89.3%, *p* = 0.001), although the objective response rate (ORR: CR + PR) showed no significant difference (*p* = 0.101). Within the new THID group, when categorized by complexity (simple vs complex) and severity (mild, moderate, severe), no significant differences were observed for CR, PR, SD, PD, ORR, or DCR (all *p* > 0.05). (Table 2 and Supplementary Table S4). Although stable disease was less common in patients with complex THID compared to those with simple THID (1.4% vs 5.4%), the difference was marginally significant (*p* = 0.052) (Table 2). Regarding adverse events, biliary injury was significantly more frequent in patients with new THID compared to those without (47% vs 14.3%, *p* = 0.002) and in the complex THID group compared to the simple THID group (40.5% vs 6.8%, *p* < 0.001). Intrahepatic metastasis was also more common in the new THID group (25.7% vs 7.1%, *p* = 0.030), although no significant differences were observed between simple and complex THID (*p* = 0.430). Similarly, portal venous thrombosis occurring after DEB-TACE showed no significant differences between simple and complex THID, as well as New THID and No New THID (*p* = 0.568 and *p* = 0.352, respectively) (Table 2 and Fig. 4). Additionally, when stratified by THID severity, no significant differences were observed in the incidence of biliary injury, intrahepatic metastasis, or portal venous thrombosis (all *p* > 0.05) (Supplementary Table S4).

**Table 2:** Comparison of simple and complex THID with treatment response and adverse events within 12 months post-DEB-TACE

Outcomes	New THID (*n* = 74)			No new THID (*n* = 28)	^‡^*p* value
	Simple THID (*n* = 29)	Complex THID (*n* = 45)	^†^*p* value		
Treatment response					
CR	11 (14.7)	13 (17.6)	0.417	13 (46.4)	0.189
PR	5 (6.8)	9 (12.2)	0.767	6 (21.4)	0.775
SD	4 (5.4)	1 (1.4)	0.052	6 (21.4)	0.033*
PD	9 (12.2)	22 (29.7)	0.128	3 (10.7)	0.002*
Objective response rate (ORR)	16 (21.6)	22 (29.7)	0.386	19 (67.9)	0.101
Disease control rate (DCR)	19 (25.7)	23 (31.1)	0.163	25 (89.3)	0.001*
Adverse events					
Biliary injury	5 (6.8)	30 (40.5)	< 0.001*	4 (14.3)	0.002*
Intrahepatic metastasis^#^	6 (8.1)	13 (8.1)	0.430	2 (7.1)	0.030*
Portal venous thrombosis	3 (4.1)	4 (5.4)	0.568	4 (14.3)	0.352

All data are presented as numbers (percentages)

The treatment response was assessed according to mRECIST

Treatment response mRECIST criteria: ORR = CR + PR vs SD + PD, DCR = CR + PR + SD vs PD

*CR* complete response, *PR* partial response, *SD* stable disease, *PD* progressive disease

^#^ Intrahepatic metastasis refers to new lesions that are discontinuous from the primary tumor, and does not include new primitive lesions

^†^ *p* indicates the difference in outcomes among different groups of THID

^‡^ *p* indicates the difference in outcomes between new THID and no new THID

* *p* values indicate a significant difference (*p* < 0.05)

**Fig. 5:**
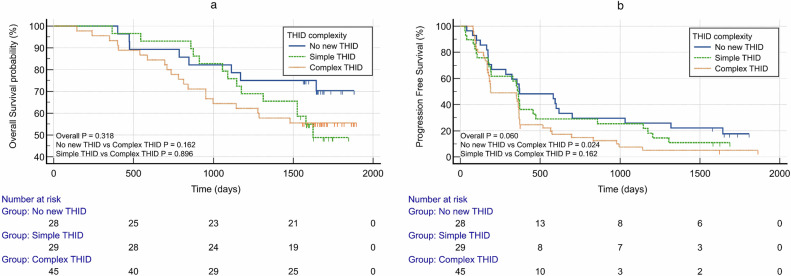
Kaplan–Meier survival curves for OS and PFS stratified by complexity of THID. **a** For OS, no significant differences were observed among the groups (overall, HR = 1.30, 95% CI: 0.89–1.89, *p* = 0.318). Pairwise comparisons also showed no significant differences: no new THID vs simple THID (*p* = 0.165), no new THID vs complex THID (*p* = 0.162), and simple THID vs complex THID (*p* = 0.896). **b** For PFS, a trend toward decreasing survival with increasing THID complexity was observed (overall *p* = 0.060), though this trend did not reach statistical significance. Pairwise comparisons showed a significant difference between no new THID and complex THID (*p* = 0.024), while comparisons between no new THID and simple THID (*p* = 0.340) and between simple THID and complex THID (*p* = 0.162) were not significant. The number at risk for each group at different time points is displayed below the graphs

**Fig. 6:**
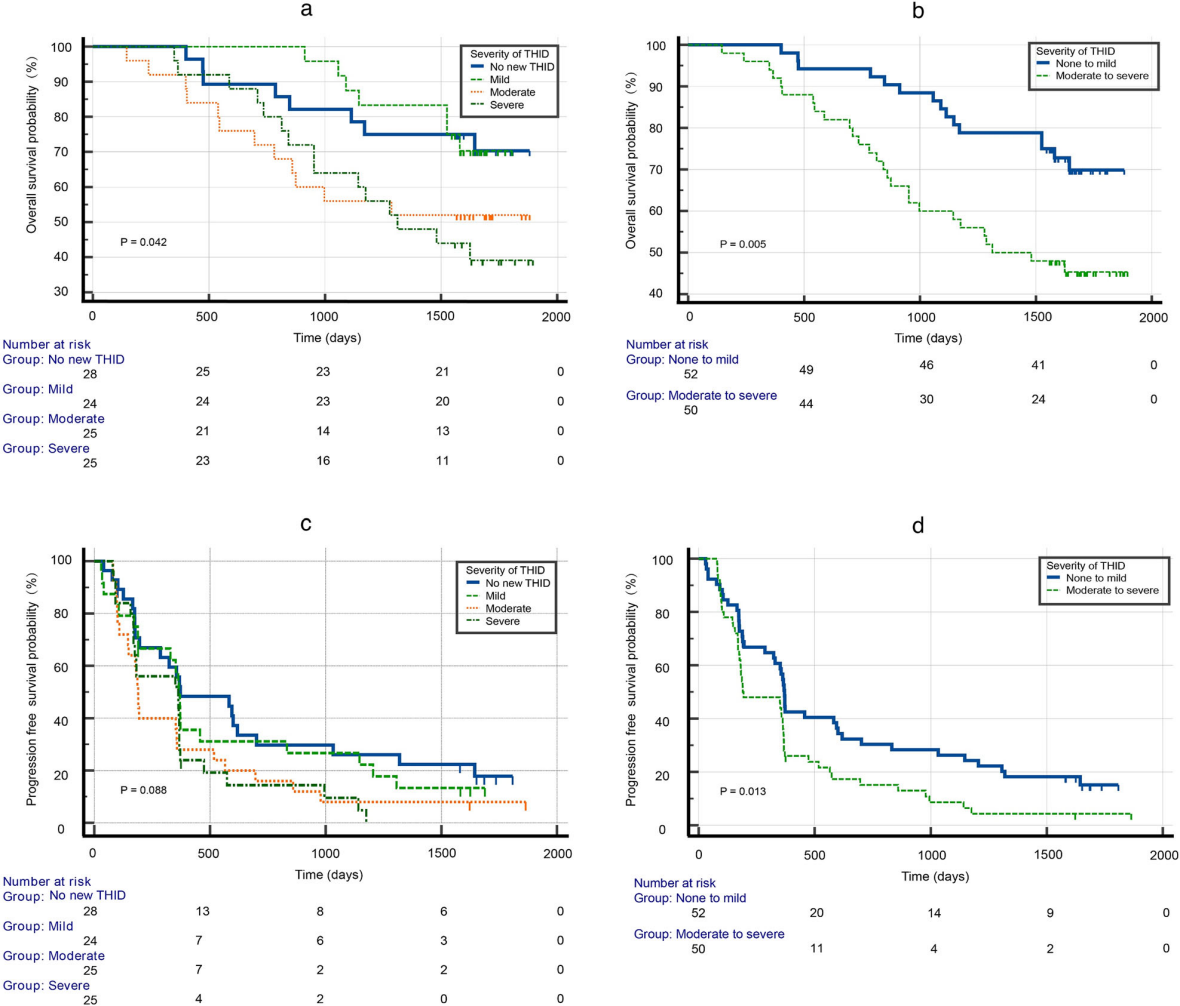
Kaplan–Meier survival curves for OS and PFS stratified by severity of THID. **a** OS stratified by severity of THID: none, mild, moderate, and severe. Patients with none-to-mild THID showed significantly better OS than those with moderate-to-severe THID (HR = 1.25, 95% CI: 1.04–1.51, *p* = 0.042). **b** OS grouped by none-to-mild vs Moderate-to-severe THID. Patients with none to mild THID had significantly better OS (HR = 2.32, 95% CI: 1.22–4.41, *p* = 0.005). **c** PFS stratified by degree of THID: none, mild, moderate, and severe. Although there is a trend towards worse PFS with more severe THID, the differences are not statistically significant (HR = 1.25, 95% CI: 1.04–1.51, *p* = 0.088). **d** PFS grouped by none-to-mild vs moderate-to-severe THID. Patients with none to mild THID had significantly better PFS (HR = 1.80, 95% CI: 1.17–2.78, *p* = 0.013)

OS and PFS were compared across THID severity and complexity using Kaplan–Meier analysis. Stratification by THID complexity (no new THID, simple THID, and complex THID) revealed no significant differences in OS among the groups (HR = 1.30, 95% CI: 0.89–1.89, *p* = 0.318). For PFS, patients with no new THID demonstrated significantly better survival compared to those with complex THID (*p* = 0.024), while comparisons between no new THID and simple THID (*p* = 0.340) and between simple and complex THID (*p* = 0.162) were not significant (Fig. 5). When stratified by severity, Survival probabilities were highest for patients with no new THID, followed by those with mild, moderate, and severe THID (HR = 1.25, 95% CI: 1.04–1.51, *p* = 0.042) (Fig. 6a). Notably, patients with none-to-mild THID had significantly better OS compared to those with moderate-to-severe THID (HR = 2.32, 95% CI: 1.22–4.41, *p* = 0.005) (Fig. 6b). For PFS, there were no significance across severity groups (HR = 1.25, 95% CI: 1.04–1.51, *p* = 0.088) (Fig. 6c). However, patients with none-to-mild THID had significantly better PFS than those with moderate-to-severe THID (HR = 1.80, 95% CI: 1.17–2.78, *p* = 0.013) (Fig. 6d). The univariate and multivariate analysis of risk factors associated with the development of THID following DEB-TACE, as summarized in Supplementary Table S5. Univariate analysis revealed that Child–Pugh class A/B (OR = 0.26, 95% CI: 0.07–0.95, *p* = 0.042), BCLC stage 0/A/B (OR = 2.15, 95% CI: 1.02–4.52, *p* = 0.044), number of tumors (OR = 3.67, 95% CI: 1.34–10.04, *p* = 0.011), Baseline THID (OR = 3.86, 95% CI: 1.21–12.29, *p* = 0.022), Total Bilirubin (OR = 0.94, 95% CI: 0.89–0.99, *p* = 0.030) and baseline prothrombin time (OR = 0.76, 95% CI: 0.61–0.96, *p* = 0.026) were significantly associated with THID development. Multivariate analysis further confirmed that several factors remained significantly associated with the development of THID; Child–Pugh class A/B (OR = 0.18, 95% CI: 0.41–0.84, *p* = 0.030), number of tumors (OR = 4.41, 95% CI: 1.38–14.07, *p* = 0.012), and Baseline THID (OR = 3.61, 95% CI: 1.07–12.17, *p* = 0.038) (Supplementary Table S5).

Interobserver agreement for THID classification was assessed using unweighted and weighted kappa statistics. For “New THID vs No New THID,” the Kappa value was 0.950 (95% CI: 0.88–1.02). For “THID complexity” (simple or complex), the Kappa value was 0.761 (95% CI: 0.67–0.86). For “THID severity” (mild, moderate, or severe), the Kappa value was 0.713 (95% CI: 0.61–0.82) (Supplementary Table S6). Partial correlation analysis evaluated the relationship between THID and clinical outcomes (biliary injury, intrahepatic metastasis) with and without adjusting for confounders (number of tumors, cumulative treatment dose, Child–Pugh score, baseline THID, BCLC stage, baseline prothrombin time, and baseline total bilirubin). Biliary injury showed stronger correlations without adjustment (*r* = 0.303, *p* = 0.002) than with adjustment (*r* = 0.244, *p* = 0.017). For intrahepatic metastasis, significance was lost after controlling for confounders (*r* = 0.104, *p* = 0.314), though unadjusted results were significant (*r* = 0.197, *p* = 0.047).

## Discussion

Our study indicates that THID following DEB-TACE is common and correlates with a high incidence of biliary injury, intrahepatic metastasis, and lower disease control rates, leading to poorer OS and PFS in patients with HCC.

In our study, up to 72.5% of patients (74/102) developed new THID post-DEB-TACE. Shimose et al also reported that DEB-TACE patients were more likely to develop arterio-portal shunt (APS), often seen as THID on imaging, compared to cTACE [18]. DEB-TACE primarily embolizes the treated arteries, leaving the peripheral portal vein patent, increasing the likelihood of APS formation and subsequent THID. Supporting this, Hien et al found that APS incidence was significantly higher after DEB-TACE than cTACE (46.0% vs 16.2%, *p* < 0.001) [19]. APS, a component of THID, is associated with hepatic artery obstruction and non-selective embolization techniques, contributing to increased subcapsular pressure, and hepatic arterial damage.

We observed a strong association between THID and biliary injury, consistent with existing literature. Pradella et al reported THID was more common in patients with biliary diseases (67.9% vs 1.78% in the control group), supporting its role as a marker for biliary complications [20]. Biliary injury post-DEB-TACE can result from ischemic damage due to embolization or direct chemical insult to vessel walls from high concentrations of doxorubicin near the DEBs [21], leading to serious complications, including biliary strictures, leaks, or secondary infections like cholangitis. Additionally, embolization strength, including bead size, number, and selectivity, plays a critical role. Non-selective embolization and larger beads may worsen biliary ischemia, increasing complications like bilomas and strictures [22]. Arai et al found THID frequency was significantly higher (*p* < 0.001) in the cholangitis group (85%) vs the control group (5%), highlighting the association between ischemic damage, biliary injury, and THID [23].

Furthermore, patients who developed THID following DEB-TACE were more likely to experience intrahepatic metastasis. Kim et al identified irregular circumferential peritumoral enhancement as a significant risk factor for MVI [24]. Miyata et al highlighted abnormalities in the corona enhancement pattern and the presence of a tumorous arterioportal shunt as indicators of portal vein tumor invasion [25]. Nishie et al further demonstrated that the size of peritumoral enhancement is a key predictor of MVI [26]. Our findings suggest an association between THID and intrahepatic metastasis, although the underlying mechanisms remain unknown. We hypothesize that DEB-TACE-induced THID may be associated with hypoxia and ischemia, which can promote metastasis by upregulating HIF-1α and VEGF, enhancing angiogenesis and tumor cell invasiveness [27]. Additionally, ischemic damage triggers an inflammatory response, releasing cytokines and growth factors that alter the tumor microenvironment, providing conditions that favor tumor cell survival and dissemination [28]. However, further research is needed to confirm this relationship and understand the underlying biological processes.

Our study found that the number of tumors, baseline THID, and Child–Pugh class A, correlated with the development of THID. A higher tumor count leads to vascular changes and blood flow obstruction, causing local hypoxia and promoting abnormal angiogenesis [29]. Additionally, multiple feeding arteries in HCC increase hepatic arterial damage, raising THID incidence post-DEB-TACE. When multiple tumors are present, more extensive embolization and higher dosages are required, amplifying arterial damage and THID occurrence [30]. THID (APS) is more common in Child–Pugh class A patients [18], likely due to better-preserved hepatic function, which may predispose them to vascular changes during DEB-TACE.

In our study, moderate-to-severe THID was associated with poorer OS and PFS, especially between patients with no new THID and those with complex THID. This relationship may stem from hepatic damage and compromised liver function associated with THID, as well as its potential connection to biliary injury and intrahepatic metastasis, which together may adversely affect patient outcomes, shortening OS and PFS. However, further research is needed to confirm these relationships and underlying mechanisms.

This study has several limitations. The retrospective, single-arm design introduces selection bias, limiting the generalizability of the findings. The sample size, although derived from multiple institutions, is still relatively small, necessitating future prospective, large-scale multicenter studies to confirm our findings. Additionally, THID can be linked to various underlying conditions, including APS, hyperemia, and other disorders, each with distinct clinical implications. Analyzing THID as a whole may overlook the specific contributions of these individual components, warranting future research to investigate them separately. Furthermore, exploring the underlying physiopathological mechanisms with histopathological evidence could help clarify the specific roles of each component of THID and its clinical significance. While we controlled for liver disease severity by including only Child–Pugh class A and B patients, incorporating metrics like the MELD score could provide deeper insights into liver function in outcomes. While our findings do not establish THID as a direct predictor of clinical outcomes, they underscore its importance as a marker associated with poorer prognoses, warranting closer attention in clinical practice.

In conclusion, THID is a common phenomenon following DEB-TACE in patients with HCC, with significant implications for clinical outcomes. This study demonstrates a strong association between the complexity and severity of THID and adverse events, particularly biliary injury and intrahepatic metastasis, which contribute to reduced OS and PFS. Furthermore, THID presence correlates with baseline patient characteristics, including the number of tumors, baseline THID, and Child–Pugh class. These findings highlight the need for careful patient selection and close monitoring during and after DEB-TACE to mitigate the risks associated with THID and improve treatment outcomes.

## Supplementary information

ELECTRONIC SUPPLEMENTARY MATERIAL

## Data Availability

The data that support the findings of this study are available from the corresponding author, Yuan-Cheng Wang, upon reasonable request.

## Abbreviations

BCLC: Barcelona clinic liver cancer
DEB-TACE: Drug-eluting bead transcatheter arterial chemoembolization
ECOG PS: Eastern cooperative oncology group performance status
HCC: Hepatocellular carcinoma
mRECIST: Modified response evaluation criteria in solid tumors
OS: Overall survival
PFS: Progression-free survival
THID: Transient hepatic intensity differences
