# Supplementary material for: Transient hepatic intensity differences: correlations with treatment outcomes and adverse events following DEB-TACE in hepatocellular carcinoma
Source: Insights Imaging. 2025 Aug 1;16:164. doi: 10.1186/s13244-025-02041-2 (PMC12316659; doi:10.1186/s13244-025-02041-2)
Supplement: Supplementary file 1 — ELECTRONIC SUPPLEMENTARY MATERIAL [file 13244_2025_2041_MOESM1_ESM.pdf]

Transient Hepatic Intensity Differences: Correlations with Treatment Outcomes and Adverse Events Following DEB-TACE in Hepatocellular Carcinoma

ELECTRONIC SUPPLEMENTARY MATERIAL

Supplementary Table S1 MR scanners with different venders in all ten centers

Center	Manufacturer	Manufacturer Model Name
1	Philips Medical Systems	Achieva
	Philips Healthcare	Achieva
2	SIEMENS	Verio
	UIH	uMR 560
	SIEMENS	Avanto
3	GE MEDICAL SYSTEMS	DISCOVERY MR750
4	Philips Medical Systems	Ingenia
	Philips Healthcare	Ingenia
5	SIEMENS	Verio
6	GE MEDICAL SYSTEMS	DISCOVERY MR750
	SIEMENS	Aera
	GE MEDICAL SYSTEMS	Optima MR360
7	SIEMENS	Skyra
	GE MEDICAL SYSTEMS	Signa HDxt
8	GE MEDICAL SYSTEMS	Signa HDxt
9	SIEMENS	Verio
	Philips Healthcare	Ingenia
	Philips Medical Systems	Ingenia
	SIEMENS	Verio
10	GE MEDICAL SYSTEMS	DISCOVERY MR750
	GE MEDICAL SYSTEMS	SIGNA Pioneer
	GE MEDICAL SYSTEMS	Signa HDxt

Supplementary Table S2 Recommended parameters for liver scans

Sequence	Repetition time (TR)/ms	Echo time (TE)/ms	Slice thickness/mm	Slices	Matrix	Field of view (FOV)/mm	Other
Coronal T2WI	2000	93	5	24	256×179	350×350	Breath hold
Transverse T2WI	2000	78	6	26	320×224	350×280	Respiratory trigger
Fast-spoiled gradient-recalled echo T1WI	200	1.54/2.91	6	26	256×192	380×285	Breath hold
DWI	1500	60 (Minimum)	6	26	128×104	350×280	Multiple breath hold; b value 0，600 s/mm^2
DCE T1WI	3.92 (Minimum)	1.39 (Minimum)	3	64	320×224	380×285	conventional extracellular and hepatocyte-specific contrast agents

Note— T1WI, T1-weighted imaging; T2WI, T2-weighted imaging; DWI, diffusion weighted imaging; DCE, dynamic contrast-enhanced.

Supplementary Table S3 Features of Transient Hepatic Differences on DCE-MR Imaging Presentation.

	Definition	Imaging Presentation
Arterioportal Shunt (APS)	The abnormal connection between the hepatic artery and portal vein leading to localized perfusion changes.[1][2]	**Non-Contrast:** Isointense or mildly hyperintense.<br>**Arterial Phase:** Hyperintense in the affected area due to arterialization, with the portal vein potentially visible.<br>**Portal Venous Phase:** May remain hyperintense or become isointense if shunting persists.<br>**Delayed Phase:** Isointense or hyperintense depending on the resolution of shunting.<br>**T2WI:** Mildly hyperintense due to altered blood flow.<br>**DWI:** May show restricted diffusion if there is significant blood flow alteration.
Hyperemia	Increased blood flow to a specific liver region, often due to inflammation or injury.[3]	**Non-Contrast:** Isointense or mildly hyperintense.<br>**Arterial Phase:** Hyperintense in the affected region.<br>Portal Venous Phase: Hyperintensity may persist or normalize depending on blood flow.<br>**Delayed Phase:** Typically returns to isointense as inflammation resolves.<br>**T2WI:** Hyperintense if associated with edema or congestion.<br>**DWI:** May show restricted diffusion if there is significant edema.
Inflammatory Reaction	Localized liver response to infection, injury, or autoimmune processes.[4]	**Non-Contrast:** Isointense or mildly hyperintense depending on severity.<br>**Arterial Phase:** Hyperintense in inflamed regions.<br>**Portal Venous Phase:** Hyperintensity may persist or normalize depending on inflammation.<br>**Delayed Phase:** Typically returns to isointense as inflammation resolves.<br>**T2WI:** Hyperintense due to edema, congestion, or inflammatory infiltrates.<br>**DWI:** Restricted diffusion is possible in areas with significant inflammation.
Peritumoral Enhancement	Circumferential or wedge-shaped high signal intensity around a tumor indicating vascular changes.[5][6]	**Non-Contrast:** Isointense or mildly hyperintense.<br>**Arterial Phase:** Hyperintense ring or halo around the tumor.<br>**Portal Venous Phase:** Peritumoral enhancement persists, and may partially wash out.<br>**Delayed Phase**: Persistent hyperintense ring or isointense depending on vascularity.<br>**T2WI:** Hyperintense if edema or inflammatory reaction is present.<br>**DWI:** May show restricted diffusion in the peritumoral area due to cellularity.

Ischemia	Reduced blood supply to a liver region, causing cell death and signal changes.[7][8]	**Non-Contrast**: Mildly hypointense or isointense.<br>**Arterial Phase**: Hypointense or non-enhancing area due to lack of arterial supply.<br>**Portal Venous Phase**: Hypointense region with poor or no enhancement.<br>**Delayed Phase**: Persistent hypointensity indicating infarcted tissue.<br>**T2WI**: Hyperintense due to edema or necrosis.<br>**DWI**: Restricted diffusion in the ischemic region.
Pseudolesions	Focal liver areas that mimic lesions, often due to variations in blood flow or fat distribution.[9][10]	**Non-Contrast:** Hypointense or isointense depending on fat content.<br>**Arterial Phase:** Variable appearance, may be hyperintense or hypointense.<br>**Portal Venous Phase:** Typically, isointense with normal liver.<br>**Delayed Phase:** Generally, returns to isointense.<br>**T2WI:** Variable, often isointense unless significant fat or fibrosis is present.<br>**DWI:** Typically, no restricted diffusion unless other pathological features are present.

References

1. Wang Q, Koniaris LG, Milgrom DP, Patel A, Hu M, Cui E, et al. CT and MRI imaging and interpretation of hepatic arterioportal shunts. Transl. Gastroenterol. Hepatol. AME Publishing Company; 2019.

2. Choi BI, Lee KH, Han JK, Lee JM. Hepatic Arterioportal Shunts: Dynamic CT and MR Features. Korean J Radiol. 2002;3:1–15.

3. Limanond P, Zimmerman P, Raman SS, Kadell BM, Lu DSK. Interpretation of CT and MRI after Radiofrequency Ablation of Hepatic Malignancies. Am J Roentgenol. 2003;181:1635–40.

4. Uemura S, Higuchi R, Yazawa T, Izumo W, Sugishita T, Morita S, et al. Impact of transient hepatic attenuation differences on computed tomography scans in the diagnosis of acute gangrenous cholecystitis. J Hepatobiliary Pancreat Sci. 2019;26:348–53.

5. Wu Y, Zhu M, Liu Y, Cao X, Zhang G, Yin L. Peritumoral Imaging Manifestations on Gd-EOB-DTPA-Enhanced MRI for Preoperative Prediction of Microvascular Invasion in Hepatocellular Carcinoma: A Systematic Review and Meta-Analysis. Front Oncol. 2022;12:1–14.

6. Wang X, Sun Y, Zhou X, Shen Z, Zhang H, Xing J, et al. Histogram peritumoral enhanced features on MRI arterial phase with extracellular contrast agent can improve prediction of microvascular invasion of hepatocellular carcinoma. Quant Imaging Med Surg. 2022;12:1372–84.

7. Kobayashi S, Kozaka K, Gabata T, Matsui O, Koda W, Okuda M, et al. Pathophysiology and imaging findings of bile duct necrosis: A rare but serious complication of transarterial therapy for liver tumors. Cancers (Basel). 2020;12:1–21.

8. Chung J, Yu JS, Chung JJ, Kim JH, Kim KW. Haemodynamic events and localised parenchymal changes following transcatheter arterial chemoembolisation for hepatic malignancy: Interpretation of imaging findings. Br J Radiol. 2010;83:71–81.

9. Kobayashi S. Hepatic pseudolesions caused by alterations in intrahepatic hemodynamics. 2021;27:7894–908.

10. Ramanathan S, Raghu V, Virmani V, Sheikh A, Al M, Tirumani S. Unveiling the unreal : Comprehensive imaging review of hepatic pseudolesions. Clin Imaging [Internet]. 2021;80:439–53. Available from: https://doi.org/10.1016/j.clinimag.2021.09.008

Supplementary Table S4. Correlation Between the THID Events, Treatment Response, and Adverse Events within 12 months Post-DEB-TACE.

Outcomes	New THID (n = 74)			
	Mild (n = 24)	Moderate (n = 25)	Severe (n = 25)	*P* value
Treatment response				
CR	9 (12.2)	9 (12.2)	6 (8.1)	0.539
PR	6 (8.1)	4 (5.4)	4 (5.4)	0.651
SD	1 (1.4)	2 (2.7)	2 (2.7)	0.827
PD	8 (10.8)	10 (13.5)	13 (17.6)	0.404
Objective response rate (CR + PR)	15 (20.3)	13 (17.6)	10 (13.5)	0.287
Disease control rate (CR + PR + SD)	16 (21.6)	15 (20.3)	12 (16.2)	0.592
Adverse Events				
Biliary Injury	8 (10.8)	12 (16.2)	15 (20.3)	0.174
Intrahepatic metastasis[#]	4 (5.4)	5 (6.7)	10 (13.5)	0.126
Portal venous thrombosis	2 (2.7)	4 (5.4)	1 (1.4)	0.341

Note: All data are presented as Number (percentage).
The treatment response was assessed according to modified Response Evaluation Criteria in Solid Tumors (mRECIST).
Treatment Response mRECIST Criteria: ORR = CR+PR vs SD+PD, DCR = CR+PR+SD vs PD.
CR- complete response, PR- partial response, SD- stable disease, PD- progressive disease.
[#]Intrahepatic metastasis refers to new lesions that are discontiguous from the primary tumor, and does not include new primitive lesions.
***P** values indicate a significant difference ($P < .05$).

Supplementary Table S5 Analysis of Risk Factors associated with the development of THID after DEB-TACE.

	Univariate		Multivariate	
Variables	OR (95% CI)	*P* value	OR (95% CI)	*P* value
Demographic				
Age (per 1 year)	1.00 (0.96-1.06)	0.727		
Sex (Male)	0.49 (0.18-1.31)	0.158		
Diabetes	1.04 (0.30-3.61)	0.941		
Hypertension	1.35 (0.48-3.83)	0.563		
Cirrhosis	0.78 (0.29-2.12)	0.637		
Child-Pugh class A/B	0.26 (0.07-0.95)	0.042	0.18 (0.41-0.84)	0.030
BCLC 0/A/B	2.15 (1.02-4.52)	0.044		
Tumor diameter baseline (cm)	0.99 (0.75-1.31)	0.965		
Number of tumors	3.67 (1.34-10.04)	0.011	4.41 (1.38-14.07)	0.012
Comorbidity				
Baseline THID	3.86 (1.21-12.29)	0.022	3.61 (1.07-12.17)	0.038
Baseline biliary injury	2.27 (0.47-10.95)	0.308		
DEB-TACE characteristics				
DEB-TACE sessions	1.24 (0.71-2.16)	0.438		
Cumulative dose	1.00 (0.99-1.01)	0.559		
Micro-sphere diameter	1.00 (0.99-1.00)	0.311		
Laboratory test				
Total Bilirubin ≤ 20.50 vs > 20.50	0.94 (0.89-0.99)	0.030		
Baseline AST ≤50 U/L vs > 50 U/L	0.98 (0.95-1.00)	0.164		
Baseline ALT ≤55 U/L vs >55 U/L	0.99 (0.97-1.02)	0.775		
Baseline alkaline phosphatase ≤129 U/L vs >129 U/L	0.99 (0.98-1.00)	0.375		
Baseline albumin ≤ 5 g/L vs > 5 g/L	0.98 (0.95-1.01)	0.228		
Baseline total protein ≤ 79 g/L vs > 79 g/L	1.00 (0.94-1.06)	0.849		
Baseline alpha-fetoprotein ≤ 200 μg/L vs > 200 μg/L	1.00 (1.00-1.00)	0.920		
Baseline prothrombin time ≤12.5 sec vs > 12.5 sec	0.76 (0.61-0.96)	0.026		

THID =Transient Hepatic Intensity Differences, AST = Aspartate aminotransferase, ALT = Alanine aminotransferase, DEB-TACE = Drug-eluting Bead Transarterial Chemoembolization, BCLC = Barcelona Clinic Liver Cancer.

Supplementary Table S6 Interobserver Agreement for THID Classification.

Assessment	Agreement[a] (95% CI)
THID Classification	
New THID vs No New THID	0.950 (0.88-1.02)
THID complexity	0.761 (0.67-0.86)
THID severity	0.713 (0.61-0.82)

Note: THID = Transient Hepatic Intensity Differences.
THID complexity (simple or complex THID).
THID severity (mild, moderate, or severe THID).
[a]Unweighted kappa coefficient for binary variables and weighted kappa coefficient for ordinal variables.
